# Is there a C-reactive protein value beyond which one should consider infection as the cause of acute heart failure?

**DOI:** 10.1186/s12872-018-0778-4

**Published:** 2018-02-27

**Authors:** Joana Pereira, Ana Ribeiro, João Ferreira-Coimbra, Isaac Barroso, João-Tiago Guimarães, Paulo Bettencourt, Patrícia Lourenço

**Affiliations:** 1 Department of Internal Medicine, São João Hospital, Porto, Portugal; 2 Department of Biochemistry, São João Hospital, Porto, Portugal; 3 Department of Clinical Pathology, São João Hospital, Porto, Portugal; 4 Unidade de Investigação e Desenvolvimento Cardiovascular do Porto, Faculty of Medicine of University of Porto, Porto, Portugal; 5 Hospital da CUF, Porto, Portugal; 6 Serviço de Medicina Interna, Hospital S. João, Alameda Professor Hernâni Monteiro, 4202-451 Porto, Portugal

**Keywords:** Acute heart failure, C-reactive protein, Infection, Cut-off

## Abstract

**Background:**

Heart Failure (HF) is a low grade inflammatory condition. High sensitivity C-reactive protein (hsCRP) is an established marker of inflammation. A cut-off value of hsCRP beyond which an infection should be sought has never been studied in HF. We aimed to determine the best hsCRP cut-off for infection prediction in acute HF.

**Methods:**

We analyzed patients included in an acute HF registry – EDIFICA (Estratificação de Doentes com InsuFIciência Cardíaca Aguda). Admission hsCRP measurement was available as part of the registry’s protocol. Patients with acute coronary syndrome as the cause of acute HF were excluded from the registry. Infection was considered according to the diagnosis registered in the discharge record. A receiver-operating characteristic (ROC) curve was used to determine the best hsCRP cut-off for infection prediction.

**Results:**

We studied 615 patients. Mean age was 76 years, 45.2% were male, 60.3% had systolic dysfunction. Median admission hsCRP was 20.3 (9.5–55.5)mg/L; in 41.6% the cause of decompensation was an infection. The area under the ROC curve for admission hsCRP in the prediction of infection was 0.79 (0.76–0.83); the best hsCRP cut-off was 25 mg/L with a sensitivity of 72.7%, specificity 77.2%, positive predictive value 69.4% and negative predictive value 79.9%. Age and elevated hsCRP independently associated with an infection as the precipitant of acute HF.

**Conclusions:**

We suggest 25 mg/L as a cut-off beyond which an infection should be sought underlying acute HF. Almost 80% of the patients with hsCRP< 25 mg/L are not infected and 69.4% of those with higher hsCRP have a concomitant infection.

## Background

Inflammation appears to play a central role in the pathophysiology of heart failure (HF) [1, 2]. Several studies have corroborated this inflammatory hypothesis, which stands that endogenous cytokine cascades are implicated in the development and progression of HF [1, 3–8]. Inflammatory biomarkers are elevated in the blood of patients with HF [9, 10]. C-reactive protein is an acute-phase protein mainly synthesized in the liver; it is the most widely used and accepted marker to assess inflammation in everyday clinical practice [9].

HF can be considered a low-grade inflammatory condition. Chronic HF patients have increased levels of high-sensitivity C-reactive protein (hsCRP), regardless of the HF aetiology, and its levels increase with the severity of the disease [4, 9, 11–14].There is also clear evidence that a systemic inflammatory response is activated in acute HF [10, 15, 16]. Most studies demonstrating this inflammatory response in acute HF excluded patients with infection or other inflammatory conditions in order to prove the independent role of inflammation in HF [10, 17]. Notwithstanding, infection is well known to frequently underlie HF decompensation [18–20].

For the general population and patients with stable coronary artery disease, an also well established low-grade inflammatory condition [8], the hsCRP cut-offs proposed for future risk of cardiovascular events are < 1 mg/L (low risk), 1-3 mg/L (intermediate risk) and > 3 mg/L (high risk). Levels > 10 mg/l should be disregarded and repeated after 2 weeks to allow any inflammation to resolve and if repeated levels persist > 10 mg/L, a non cardiovascular aetiology for such hsCRP elevation should be considered [21, 22]. No hsCRP value beyond which an inflammatory or infectious condition has to be excluded as ever been proposed for HF, neither acute, nor chronic. The knowledge of such a cut-off would be particularly useful in the acute setting in which an infection is often the precipitant of HF decompensation.

The aim of our work was to determine the best cut-off value of hsCRP to predict infection in patients presenting with acute HF.

## Methods

We conducted a retrospective cohort study in a group of patients that had been included in an acute HF registry - EDIFICA (Estratificação de Doentes com InsuFIciência Cardíaca Aguda) - that took place in the Internal Medicine department of São João Hospital Center between January 2009 and December 2010. Patients eligible for inclusion were all patients admitted to our department with the primary diagnosis of acute HF during such period; both de novo and worsening chronic HF were eligible. The 2008 European Society of Cardiology guidelines were used for the diagnosis of HF [20] and both patients with reduced and preserved ejection fraction were included; patients with ejection fraction≥50% were considered to have HF with preserved ejection fraction (HFpEF). Patients with acute coronary syndrome as the cause of acute HF were excluded from the registry. A complete physical examination at admission and in the discharge day was performed to all patients. A 12-lead electrocardiogram was also performed at admission. As part of the registry’s protocol, patients were drawn a venous blood sample within the first 48 h of hospital admission. An echocardiogram was performed to all patients during hospitalization, images were obtained with a standard ultrasound equipment (System 6, GE Vingmed, Horten, Norway) with a 2.5-MHz probe. The patient’s treatment strategy, timing of discharge and discharge medication were at the discretion of the attending physician. The attending physicians were aware of the ongoing registry.

B-type natriuretic peptide (BNP) determination is a routine laboratory procedure in our hospital; an Abbott chemiluminescentmicroparticle immunoassay (2-step immunoassay) is used. Serum creatinine was measured using conventional methods with an Olympus AU5400® automated clinical chemistry analyzer Beckman-Coulter®. Haemoglobin was obtained using an automated blood counter Sysmex® XE-5000 (Hyogo, Japan), differential blood counts were also performed in accordance with routine clinical practice.

Serum hsCRP was assayed using particle-enhanced immunonephelometric assays on a BN™II laser nephelometer (Siemens, Erlangen, Germany). The manufacturer claims, for three different hs-CRP concentrations, 2.39 mg/L, 6.50 mg/L, 7.72 mg/L, intra-assay coefficients of variation (CV) of 5.2%, 5.0% and 4.6%, respectively, and inter-assay CV of 5.2%, 5.7% and 5.4%. The normal range of hsCRP is < 3.00 mg/L.

The glomerular filtration rate was estimated using the Modification of Diet in Renal Disease (MDRD) Study Equation.

All patients provided written informed consent to participate in the study. The registry’s protocol conformed to the ethical guidelines of the declaration of Helsinki and it was approved by the local ethics committee.

**Fig. 1:**
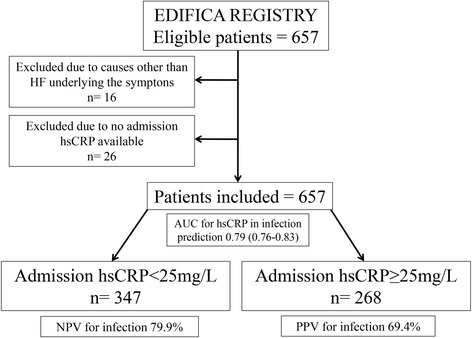
Study flow chart and main results

A retrospective analysis was conducted in this patient cohort to determine the admission hsCRP beyond which an infectious condition should be sought. Patients with missing data regarding admission hsCRP (26 patients) were excluded from the analysis. The study flow chart is depicted in Fig. 1.

An infectious condition at admission and underlying acute HF was considered according to the discharge diagnosis list as judge by the attending physician and also according to the information in the discharge record.

### Statistical analysis

We used a receiver-operating characteristic (ROC) curve to define the best hsCRP cut-off for association with an infectious condition. The area under the ROC curve (AUC) was determined.

Patients with an infectious condition underlying acute HF and those without concomitant infection were compared; and patients with hsCRP< 25 mg/L and those with ≥25 mg/L were also compared: Chi square test for categorical variables, Student’s t test to compare continuous variables and a Mann-Whitney U test when continuous variables had a highly skewed distribution. Variables independently associated with infection upon admission and underlying HF decompensation and variables independently associated with elevated hsCRP were assessed using multivariate logistic regression analysis. Models were built taking into consideration variables with a differential distribution between the groups compared.

The *p* value considered for statistical significance was 0.05. Data was stored and analyzed using SPSS® software (IBM corp, Armonk, NY, version 20.0).

## Results

We studied 615 acute HF patients (Fig. 1). Mean patient’s age was 76 (±12) years, 278 (45.2%) patients were male, 60.3% had left ventricular systolic dysfunction and 42.4% (261 patients) had ejection fraction< 35%. The percentage of patients with devices was modest, 7.3% of the patients with severe dysfunction; however we were dealing with an acute HF cohort of old and very old fragile patients. Median (interquartile range) admission hsCRP was 20.3 (9.5–55.5) mg/L. In 256 (41.6%) patients, an infection was considered as the factor precipitating or decompensating HF [68.0% had a respiratory infection, 15.2% a urinary infection, 7.8% a cutaneous infection, in 2.0% of the patients there were other, rarer, foci of infection (abdominal, cardiovascular, bones/joints), and in 7.0% the infectious focus remained unknown]. Table 1 compares patients according to infection status upon admission. Apart an expected higher admission hsCRP (median value 53.1 mg/L in infected patients against 12.8 mg/L median value in non-infected) and neutrophil count, patients with a concomitant infection at admission were more often older women, with higher proportion of patients with HF with preserved ejection fraction and were more often admitted with lower systolic blood pressure, lower haemoglobin and worse renal function. Acute HF patients with a concomitant infection less frequently had coronary artery disease and there was also a non-significant trend for them to have higher BNP levels and less often to have diabetes.

**Table 1:** Comparison between acute HF patients with and without an infectious condition underlying cardiac decompensation

Characteristics	Non-infected patients (*n* = 359)	Infected patients (*n* = 256)	*p*-value
Male sex, n (%)	175 (48.7)	103 (40.2)	0.04
Age, mean (SD)	75 (12)	79 (11)	< 0.001
Atrial fibrillation, n (%)	173 (48.2)	107 (41.8)	0.12
Diabetes *mellitus*, n (%)	159 (44.3)	94 (36.7)	0.06
Coronary heart disease, n (%)	159 (44.4)	92 (36.0)	0.04
Admission NYHA IV (vs II/III)	209 (58.2)	162 (63.3)	0.29
Admission heart rate (bpm), mean (SD)	90 (25)	88 (21)	0.34
Admission systolic blood pressure (mmHg), mean (SD)	136 (31)	130 (28)	0.01
Left ventricular systolic dysfunction, n (%)	234 (65.1)	137 (53.5)	0.003
Haemoglobin (mmol/L), mean (SD)	7.4 (1.4)	7.1 (1.3)	0.001
Leukocytes (cells/μL)	7934 (3056)	9704 (4122)	< 0.001
Neutrophils (cells/μL)	5774 (2511)	7733 (3815)	< 0.001
Monocyte-to-Lymphocyte Ratio, mean (SD)	0.54 (0.37)	0.65 (0.40)	0.001
Glomerular filtration rate (mL/min/1.73m^2^)	48.3 (20.9)	44.9 (20.7)	0.05
C-reactive protein (mg/L), median (IQR)	12.8 (7.1–24.2)	53.1 (22.8–116.0)	< 0.001
C-reactive protein ≥25 mg/L	82 (22.8)	186 (72.7)	< 0.001
BNP (pmol/L), median (IQR)	458.8 (257.2–783.5)	547.4 (293.7–858.1)	0.08
Acetylsalicylic acid, n (%)	174 (48.5)	119 (46.5)	0.65
Statin, n (%)	188 (52.4)	120 (46.9)	0.19
1-year death	130 (36.2)	102 (39.8)	0.36

*BNP* B-type natriuretic peptide, *hsCRP* high sensitivity C-reactive protein, *IQR* interquartile range, *NYHA* New York Heart Association, *SD* standard deviation

**Fig. 2:**
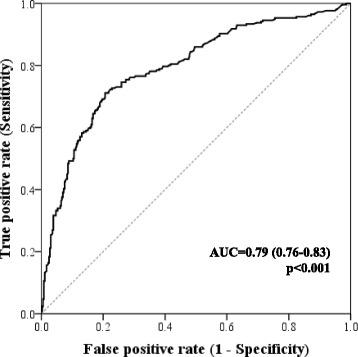
Receiver-operating characteristic (ROC) curve of the ability of admission hsCRP for infection prediction in patients hospitalized with acute HF. The AUC is of 0.79 (0.76–0.83), *p* < 0.001

The AUC for hsCRP in the prediction of infection as the cause underlying cardiac decompensation was of 0.79 (0.76–0.83), *p* < 0.001. Figure 2 depicts the ROC curve of the association of hsCRP with an infectious condition. The hsCRP value of 25 mg/L corresponded to the best cut-off for associated infection prediction. Using the cut-off of 25 mg/L, infection would be detected with a sensitivity of 72.7% and a specificity of 77.2%. The positive predictive value for infection when hsCRP ≥25 mg/L was 69.4% and the negative predictive value for infection when hsCRP< 25 mg/L was 79.9% (Fig. 1). If the classically accepted cut-off of 10 mg/L used in the general population and stable coronary heart disease was to be applied in this study sample of acute HF patients, the sensitivity would be of 91.4% and the specificity of 38.7%; this 10 mg/L cut-off would show a higher negative predictive value of 86.3%, however the positive predicted value would result unacceptably low – 51.5%.

Table 2 shows the comparison between patients admitted with hsCRP< 25 mg/L and those with hsCRP≥25 mg/L. Patients with higher admission hsCRP more often had an infectious condition underlying acute HF, were more often male, with lower admission systolic blood pressure, lower admission haemoglobin, higher BNP, and expectedly higher neutrophil count.

**Table 2:** Comparison between acute HF patients admitted with hsCRP below and above 25 mg/L

Characteristics	hsCRP< 25 mg/L (*n* = 347)	hsCRP≥25 mg/L (*n* = 268)	p-value
Male sex, n (%)	144 (41.5)	134 (50.0)	0.04
Age, mean (SD)	76 (12)	77 (12)	0.53
Atrial fibrillation, n (%)	159 (45.8)	121 (45.1)	0.88
Diabetes *mellitus*, n (%)	149 (42.9)	104 (38.8)	0.30
Coronary heart disease, n (%)	143 (41.2)	108 (40.3)	0.83
Admission NYHA IV (vs II/III)	207 (59.6)	164 (61.2)	0.87
Admission heart rate (bpm), mean (SD)	88 (24)	89 (23)	0.58
Admission systolic blood pressure (mmHg), mean (SD)	137 (29)	130 (29)	0.01
Left ventricular systolic dysfunction, n (%)	215 (62.0)	156 (58.2)	0.22
Haemoglobin (mmol/L), mean (SD)	7.4 (1.3)	7.1 (1.3)	< 0.001
Leukocytes (cells/μL)	7918 (3172)	9645 (3972)	< 0.001
Neutrophils (cells/μL)	5861 (2830)	7534 (3543)	< 0.001
Monocyte-to-Lymphocyte Ratio, mean (SD)	0.53 (0.36)	0.67 (0.40)	< 0.001
Glomerular filtration rate (mL/min/1.73m^2^)	48.0 (20.7)	45.4 (21.0)	0.13
Infectious condition at admission, n (%)	70 (20.2)	186 (69.4)	< 0.001
BNP (pmol/L), median (IQR)	446.8 (252.2–797.0)	549.0 (304.4–848.8)	0.05
Acetylsalicylic acid, n (%)	167 (48.1)	126 (47.0)	0.69
Statin, n (%)	165 (47.6)	143 (53.4)	0.19
1-year death	130 (37.5)	102 (38.1)	0.88

*BNP* B-type natriuretic peptide, *hsCRP* high sensitivity C-reactive protein, *IQR* interquartile range, *NYHA* New York Heart Association, *SD* standard deviation

Despite the different distribution of variables according to the coexistence of infection upon admission, the only variables independently associated with an infectious condition complicating acute HF were age, neutrophil count and hsCRP, both analysed as a continuous and as a categorical variable (cut-off 25 mg/L). Table 3 shows the multivariate regression models for infection prediction in acute HF, with hsCRP analysed as a continuous and as a categorical variable. Increasing age, neutrophil count and admission hsCRP were independently associated with the coexistence of an infection upon admission with acute HF. Patients admitted with acute HF who presented with a hsCRP≥25 mg/L had an approximately 8-fold higher risk of having an infection as the cause of cardiac decompensation than when admission hsCRP was lower.

**Table 3:** Predictors of an infectious condition underlying decompensated HF: multivariate models

	OR (95% CI)	*P*-value	Wald
Age, per year	1.03 (1.01–1.05)	0.01	6.60
hsCRP≥25 mg/L	8.05 (5.24–12.36)	< 0.001	90.82
Male sex	0.68 (0.43–1.08)	0.10	2.72
Diabetes *mellitus*	0.89 (0.72–1.10)	0.27	1.22
Coronary heart disease	0.73 (0.47–1.12)	0.15	2.05
Admission systolic blood pressure, per mmHg	1.00 (0.99–1.00)	0.18	1.77
Haemoglobin, per mmol/L	0.87 (0.74–1.03)	0.11	2.53
BNP per 100 pmol/L	1.02 (0.98–1.06)	0.30	1.09
Leukocytes per 1000 cells/μL	0.90 (0.76–1.06)	0.21	1.58
Neutrophils per 1000 cells/μL	1.32 (1.09–1.60)	0.005	7.88
Monocyte-to-Lymphocyte Ratio	0.96 (0.53–1.74)	0.88	0.02
Glomerular filtration rate per 10 mL/min/1.73 m2	1.08 (0.96–1.21)	0.22	1.48
Left ventricular systolic dysfunction	0.76 (0.48–1.22)	0.26	1.28
	OR (95% CI)	*P*-value	
Age, per year	1.02 (1.00–1.04)	0.02	5.51
hsCRP per 1 mg/L	1.02 (1.01–1.02)	< 0.001	44.85
Male sex	0.84 (0.54–1.30)	0.44	0.60
Diabetes *mellitus*	0.90 (0.73–1.10)	0.31	1.02
Coronary heart disease	0.77 (0.51–1.17)	0.23	1.46
Admission systolic blood pressure, per mmHg	0.99 (0.99–1.00)	0.11	2.51
Haemoglobin, per mmol/L	0.86 (0.73–1.02)	0.08	3.17
BNP per 100 pmol/L	1.01 (0.98–1.05)	0.48	0.50
Leuckoytes per 1000 cells/μL	0.88 (0.72–1.08)	0.22	1.51
Neutrophils per 1000 cells/μL	1.33 (1.06–1.66)	0.01	6.31
Monocyte-to-Lymphocyte Ratio	0.84 (0.45–1.55)	0.57	0.32
Glomerular filtration rate per 10 mL/min/1.73m^2^	1.05 (0.94–1.17)	0.41	0.67
Left ventricular systolic dysfunction	0.77 (0.49–1.20)	0.25	1.34

*BNP* B-type natriuretic peptide, *CI* Confidence Interval, *hsCRP* high sensitivity C-reactive protein, *OR* Odds Ratio

Independent predictors of elevated hsCRP at admission (≥25 mg/L) were male gender (OR = 1.87, 95% CI: 1.20–2.91) and haemoglobin (OR = 0.76, 95% CI: 0.64–0.90 per each mmol/L increase). An infectious condition was also an independent predictor of elevated hsCRP with a 8-fold higher risk. Adjustments were made accounting for age, diabetes, coronary artery disease, admission systolic blood pressure, admission glomerular filtration rate, BNP, leukocyte and neutrophil counts, monocyte to linfocyte ratio and left ventricular systolic dysfunction (data not shown).

## Discussion

Our results add some important findings to the current knowledge on the role of hsCRP and inflammation in acute HF. In our 615 patients’ cohort, 25 mg/L value appears to be the best hsCRP cut-off for prediction of concomitant infection in the acute HF setting. In 72.7% of the patients presenting with acute HF, in whom an underlying infection decompensates HF, the hsCRP value was ≥25 mg/L. On the other hand, if a patient presented with acute HF and had an admission hsCRP ≥25 mg/L he had an almost 70% probability of having an infectious condition causing the cardiac decompensation. Our results also suggest that patients hospital-admitted with acute HF that have a hsCRP< 25 mg/L we are almost 80% certain that no infection complicates the clinical scenario.

In accordance with previous publications [18, 19, 23], we report a high prevalence of concomitant infection in patients presenting with acute HF and reinforce infection as an important precipitant of acute HF. Procalcitonin, another inflammatory biomarker, was found to be higher in patients with HF in comparison with control subjects and patients in whom acute HF and infection coexisted presented even higher levels of procalcitonin [24]. The recognition that an often life threatening infection precipitates acute HF and that the diagnosis of superimposed infections is sometimes difficult in this context has led to the development of strategies to more accurately identify coexistent infection. Procalcitonin has emerged as a promising tool for the early and accurate diagnosis of pneumonia and for guiding antibiotic therapy in acute HF patients [25, 26]. However, hsCRP is an inflammatory marker more widely available, more extensively studied, and much more familiar to physicians treating HF patients. Moreover, the AUC reported for procalcitonin in the diagnosis of pneumonia was of 0.72, which is lower than the one we report for the association of hsCRP with an infection underlying HF decompensation – AUC = 0.79 [27]. HsCRP has additional recognized prognostic information in the HF context rendering it a perhaps more useful biomarker in acute HF [17, 28–30].

Despite the differences between groups of patients with hsCRP below and above the 25 mg/L cut-off, the only variables independently associated with higher hsCRP were male gender, lower hemoglobin and concomitant infection. The relationship between gender and hsCRP is still controversial; however, inflammatory activation and the role of inflammation are clearly gender influenced. Depending on the population studied higher hsCRP values either in male or in female have been reported [31, 32]. Anemia is a common comorbidity in inflammatory conditions – anemia of chronic diseases [33]. Despite differences between groups of acute HF patients with and without a concomitant infection upon admission, the only independent predictors of infection were older age, higher neutrophil count and higher hsCRP. Older patients become progressively immune incompetent in one hand and have higher comorbidity burden in the other hand and, therefore, are more prone to infectious complications of their chronic diseases [34]. HsCRP is a well studied and accepted non-specific biomarker of inflammatory and infectious conditions [35]. Of note that the monocyte-to-lymphocyte ratio was not independently associated with the coexistence of an infectious condition; this eventually reflects the fact that the monocyte-to-lymphocyte ratio is probably more associated with chronic inflammatory burden and cardiovascular risk [36, 37] and less a mirror of acute changes in response to infection.

As a low-grade inflammatory disorder we would expect patients with HF, and particularly those with acute HF, to present with elevated hsCRP. In fact, in our patient population, those with acute HF and no infectious condition had a median hsCRP value at admission of 12.8 mg/L. It is important to remember that inflammatory and oxidative acute and chronic conditions may affect the electrophysiological functions, and the electrical/anatomical properties of cardiac function [38]. This could have been addressed by device interrogation; however, the number of patients with devices was low. This issue is even more pertinent if we consider this is a cohort of old HF patients and that 41.1% of them were diabetic, factors known to affect patients’ response to device implantation and HF prognosis [39, 40].

The prognostic significance of hsCRP is documented in HF, mainly in the chronic context [9, 13, 41]; but also in the acute setting [29]. The interpretation of hsCRP in acute HF patients may be more intricate precisely because infectious intercurrences frequently underlie the HF worsening and hsCRP in a non-specific marker of inflammation. Part of the acute HF treatment implies addressing the decompensating factor and a clear recognition of an infection and its treatment may be crucial [42]. This is even more important if we consider that clinical manifestations of infection can be very mild and atypical in this group of classically old and very old patients.

Previous studies have shown that hsCRP has a prognostic role in acute HF patients and that higher levels of this acute-phase protein are associated with higher mortality [29]. Most studies that demonstrated an inflammatory response in acute HF excluded patients with concurrent infection or other inflammatory conditions in order to prove the independent role of inflammation in HF [10, 17]. The exact value that could help distinguishing patients with elevated hsCRP in relation with the inflammatory process underlying HF and those with elevated hsCRP due to a concomitant infectious process was never studied in HF. This is important, particularly in acute HF, not only to help defining the treatment strategy but also for clinicians to be able to interpret the prognostic value of hsCRP.

To the best of our knowledge this is the first study proposing a cut-off value beyond which an infection is highly prevalent in acute HF patients and therefore needs to be sought. The existence of such a cut-off can eventually help clinicians treating acute HF patients in their everyday clinical practice, representing a major advantage in the management of these patients with unquestionable clinical implications.

We propose the cut-off of 25 mg/L as the one beyond which hsCRP elevation should be considered as not totally attributable to low grade inflammation in acute HF. This value is higher than the arbitrary value of 10 mg/L accepted for the general population and patients with stable coronary artery disease.

This 25 mg/L cut-off value for hsCRP that we propose is grown out of a relatively large population of “real world” acute HF patients. The fact that it is significantly higher than the one for the general population is not totally unexpected since acute HF patients are experiencing a decompensation/exacerbation of an inflammatory condition. The use of the classical cut-off of 10 mg/L would have an elevated sensitivity (over 90%), but an also extremely elevated false positive rate of 61.3% and a low positive predictive value with only approximately half of the patients having an infectious condition when hsCRP value at acute HF presentation was ≥10 mg/L.

Our study has some limitations that need to be noted. The single center nature of the study implies generalisability concerns. The retrospective nature of the study has known inherent problems, namely concerning the availability and quality of the data collected. A treatment and general approach bias related to the fact that physicians treating HF patients were aware of the ongoing registry should also be kept in mind. It would have also been interesting to have included in the multivariate models other inflammatory markers, such as cytokines or TNF-α, however, no such data were available. Perhaps the most important setback comes from the definition of infection. We relied on the clinical evaluation of the attending physician and its registry in the discharge diagnosis list or in the discharge record. Criteria could be different between the physicians but, consulting the discharge notes, there were clinical and laboratory or radiologic criteria to support the diagnosis of infection.

Apart these limitations we were able to show that in the setting of acute HF, clinicians can rely on hsCRP values to adequately diagnose infection as the trigger of the decompensation and to treat it in accordance. Acute HF patients represent a group with a low grade inflammatory condition experiencing an episode of decompensation and an elevation of hsCRP is already expected. We have documented such hsCRP elevation in non-infected patients as well as an even higher elevation in infected ones. We have as well documented the ability of hsCRP to predict a concurrent infectious condition in acute HF decompensations.

## Conclusions

In patients with acute HF presenting with a hsCRP≥25 mg/L, an infectious condition complicating or underlying cardiac decompensation should be sought. Almost 80% of the patients with hsCRP< 25 mg/L are not infected and 69.4% of those with higher hsCRP have a concomitant infection.

## Abbreviations

AUC: Area under the curve
BNP: B-type natriuretic peptide
BPM: Beats per minute
CI: Confidence Interval
HF: Heart Failure
HFpEF: Heart Failure with preserved ejection fraction
hsCRP: High sensitivity C-reactive protein
IQR: Interquartile range
NYHA: New York Heart Association
OR: Odds Ratio
ROC: Receiver-operating characteristic
SD: Standard deviation
